# Sperm Cryopreservation Today: Approaches, Efficiency, and Pitfalls

**DOI:** 10.3390/cimb45060300

**Published:** 2023-05-29

**Authors:** Sanja Ozimic, Helena Ban-Frangez, Martin Stimpfel

**Affiliations:** 1 Department of Human Reproduction, Division of Obstetrics and Gynecology, University Medical Centre Ljubljana, 1000 Ljubljana, Slovenia; sanja.ozimic@kclj.si (S.O.); helena.ban@kclj.si (H.B.-F.); 2 Faculty of Medicine, University of Ljubljana, 1000 Ljubljana, Slovenia

**Keywords:** cryopreservation, cryodamage, human spermatozoa, cryoprotectant, slow freezing, vitrification

## Abstract

The cryopreservation of human spermatozoa has been an option for patients undergoing chemo or radiotherapies since the late 1950s. Presently, there are different techniques for the cryopreservation of spermatozoa. The most commonly used techniques are programmable slow freezing and freezing on liquid nitrogen vapors, while the use of vitrification is still not accepted as clinically relevant. Although there have been many improvements, the ideal technique for achieving better post-thaw sperm quality continues to be a mystery. A major obstacle during cryopreservation is the formation of intracellular ice crystals. Cryodamage generated by cryopreservation causes structural and molecular alterations in spermatozoa. Injuries can happen because of oxidative stress, temperature stress, and osmotic stress, which then result in changes in the plasma membrane fluidity, motility, viability, and DNA integrity of the spermatozoa. To prevent cryodamage as much as possible, cryoprotectants are added, and in some clinical trial cases, even antioxidants that may improve post-thaw sperm quality are added. This review discusses cryopreservation techniques, cryodamage on molecular and structural levels, and cryoprotectants. It provides a comparison of cryopreservation techniques and describes recent advances in those techniques.

## 1. Background and General Principles of Cryopreservation

Nowadays, the cryopreservation of gametes, embryos, and tissues (ovarian and testicular) can be found in many different fields. Cryopreservation is also a well-known, routine technique used in assisted reproduction to preserve genetic material for decades [1]. In the case of human semen, this approach is very valuable and is used in patients undergoing chemo or radiotherapies and patients with other auto-immune diseases; it can also be used in patients with severe oligozoospermia and ejaculatory disorder, and we must not forget the cryopreservation of donor semen [2]. The freezing of human semen was first noted in 1776 by Spallanzani, an Italian priest who described the effect of low temperatures on the motility of spermatozoa by observing the effect of snow on human sperm [3]. Since then, there have been many possibilities described for the establishment of biobanks for cryopreserved human semen and how useful these banks would be. The first individual to discuss sperm banks was the Italian neurologist, anthropologist, and physiologist Paolo Mantegazza. In 1886, his exact words were: “It might even be that a husband who has died on the battle-field can fecundate his own wife after he has been reduced to a corpse and produce legitimate children after his death” [4]. After this, science was fascinated by how spermatozoa behave at low temperatures, and a major breakthrough happened in 1950 with pregnancies achieved via insemination with frozen sperm [5]. The possibility of cryopreserving human spermatozoa has existed for over 80 years now, and empirical methods are still used today; however, in the current period, quite a few improvements have been made to the procedure. A common goal of all techniques used is to achieve the highest post-thaw cell survival possible because the cells face many obstacles during freezing, and cryoinjuries can happen. A major obstacle is the formation of intracellular ice crystals. We know that liquid water is essential to the function of living cells, but its solidification can be lethal. To bypass those obstacles as much possible and for cryopreservation to be successful, some standard key steps must be followed. The first step is storage in liquid nitrogen (−196 °C). At this temperature, there is insufficient thermal energy, and the processes of cell metabolism cease [6]. Then, before freezing, we must add a cryoprotective agent to the samples that reduces the amount of intracellular water and prevents intracellular ice crystals [7]. The third and fourth steps are thawing the samples and removing the cryoprotectant from the cells after thawing. All cryopreservation techniques, which are slow freezing, rapid freezing, and vitrification (which can also be called ultrarapid freezing), do include those steps, but we can note some differences between the protocols. The most significant difference is the speed of freezing and thawing, followed by the use of a cryoprotectant and its concentration [8]. For the record, the temperature drop during slow freezing occurs at a rate from 0.5 °C to 0 °C per minute, and during vitrification, the temperature drops by hundreds or even thousands of °C per minute [7,9]. Studies agree that most cryoinjuries happen due to freezing the cells at an incorrect freezing speed, whether too slow or too fast. If the speed is too fast, water is not removed quickly enough, and intracellular ice crystals are produced that can damage cell organelles; if the speed is too slow, the cells eliminate water rapidly and dehydrate [6,10]. Neither option is good for cell survival; both too-low or too-high cooling rates can kill cells, so an optimal cooling rate should exist between the ”low“ and “high“ rates. Known cryodamage, which can happen when freezing spermatozoa, can be observed at different cellular functions and levels, such as (1) a decrease in sperm motility and viability, (2) a decrease in mitochondrial activity rates, (3) a decrease in DNA integrity, and (4) an increase in the production of reactive oxygen species (ROS) [11]. These are also basic parameters used in the evaluation of sperm quality after the freezing and warming processes. Cryoinjuries can be avoided with cryoprotective agents (CPAs). Their task is to reduce the stress produced by cryopreservation techniques [12]. Usable CPAs are permeable (DMSO, glycerol, ethylene glycol, and 1,2-propanediol) and non-permeable (glucose, sucrose, and trehalose). Permeable CPAs easily cross the cell membrane and cause water to leave the cell, principally slowly via simple diffusion or, in some cases, rapidly by facilitating diffusion via channels [13]. Non-permeable CPAs do not cross the cell membrane: they increase concentration outside the cell and cause water to leave the cell [14].

Cryopreservation is a very complex procedure because it covers various protocols, freezing carriers, and cryoprotective agents [15]. Several studies are seeking the most suitable technique to achieve the highest rate of cryosurvival possible. Therefore, in this review, we discuss the basic facts of the cryopreservation of spermatozoa and pay attention to all cryopreservation techniques. In summary, we talk through the positive and negative effects cryoprotective techniques have on spermatozoa, including on a molecular level, to find the right balance to achieve favorable and optimal results for assisted reproduction techniques.

## 2. The Impact of Cryopreservation on Human Spermatozoa (Cryodamage)

Cryopreservation is a process in which spermatozoa undergo dramatic changes which can call their fertilizing potential into question. These changes are related to two main factors: the formation of ice crystals and the addition and removal of CPAs [16]. To retain several features necessary for egg fertilization, it is recommended to know the complete sperm physiology during cryopreservation and to consider the environment to which we expose spermatozoa during cryopreservation and how this environment is reshaped at different rates of cooling [17]. The most important and most commonly reported features are motility, viability, acrosomal integrity, and DNA content [18]. Spermatozoa likely have huge potential compared to other cells because they have adequate characteristics (small cells with a large surface area) to reduce potential damage due to cryopreservation [19]. According to researchers, the most well-known cryoinjuries are ruined DNA integrity (DNA fragmentation, chromatin decondensation, and variations in acrosomal state), reductions in motility and viability, and changes in the plasma membrane’s fluidity and integrity [20].

### 2.1. Plasma Membrane Fluidity and Spermatozoa Viability

Biomembranes form cells, separate the outside and inside of an organism, and enable living organisms to generate energy. They are composed of lipid bilayers and have selective permeability, which means that they control which substances enter and leave and the flow of messages between cells in the form of chemical and electrical signals [21]. Some of the membrane components are proteins, whose tasks include (1) relaying signals between the cell and its environment, (2) moving molecules and ions across the membrane, and (3) involvement in the immune response and (4) enzyme activities [22]. The other essential components are lipids, which work as energy storage molecules, chemical identifiers, and signaling molecules [23]. The physical properties of lipids in biological membranes are highly sensitive to changes in temperature. Two main steps in cryopreservation (freezing and thawing) are based on sudden temperature changes, and because the membranes in spermatozoa are rich in fatty acids, they are the primary sites of cryoinjury. Researchers claim that membrane viability must be taken into consideration because spermatozoa are capable of fertilization only with highly regulated membrane organization. Membranes are in a fluid phase under normal conditions, but when extreme temperature changes occur, the membrane’s consistency breaks, and its components mix and lose conformation. Every component of the membrane (proteins, lipids, and sterols) has unique characteristics which are irreversible, and changes in these characteristics due to temperature stress are the main reasons for the structural and functional loss of plasma membrane integrity [6,24]. Aside from temperature stress, membrane destabilization also occurs because of the large volume changes that are linked with cryoprotectant and water movements and osmotic stress. Differences in the sperm membrane caused by cryopreservation occur in its membrane hydraulic permeability, phospholipid composition, osmotic tolerance limits, and cholesterol limits. All of these changes are thought to be involved in the loss of permeability [6,25]. Giraud et al. [26] proposed the hypothesis that the fluidity of the sperm membrane reflects the physiological status of the membrane, and it is very important for spermatozoa to be able to restore their membranes after freezing and thawing. They took 20 semen samples from normozoospermic men and focused on sperm motility and viability and on an assessment of fluidity. They created an eosin test dye and found that there was a decline in viable spermatozoa that was related to membrane permeability, meaning that membrane fluidity decreased following cryopreservation, dynamics were changed, and membrane functions that depend upon membrane fluidity were also changed; thus, we can say that all these factors together caused the loss of barrier function. This was also confirmed by James et al. [9], who were preserving the integrity of the plasma membrane by measuring lipid diffusion in different regions of spermatozoa. They found that lipid diffusion was decreased in the acrosome, midpiece, and postacrosome, indicating that membrane fluidity was changed.

One of the basic parameters in sperm analysis is viability, which is useful when the motility is very low (5–10%) to determine if nonmotile sperm are dead or alive. Two methods are used in laboratories: the eosin test and the HOS test. A common point of both tests is the focus on sperm membrane integrity. During an eosin test, live sperm are able to resist the absorption of certain dyes; during an HOS test, water enters the cytoplasm and causes the tail to swell. The impact of cryopreservation on the sperm membrane is well known, and many studies agree that frozen–thawed semen is less fertile than fresh semen because of reduced sperm motility and a lower number of viable spermatozoa [27,28]; however, it does not seem to negatively influence the live birth rate [29].

### 2.2. Motility

A basic sperm analysis must be performed to determine if the sperm are capable of first reaching and then fertilizing an egg. There must be enough progressively motile spermatozoa in the ejaculate for at least one spermatozoon to reach an oocyte. The determination of sperm motility is biologically and clinically important because the presence or absence of rapid, progressive spermatozoa gives us better prognostic information for ART [30,31]. Sperm cells are rich in mitochondria because a constant supply of energy is required for their motility, and mitochondrial damage during cryopreservation processes is linked with a loss of membrane permeability. This confirms the fact that cryopreservation affects motility because of mitochondrial damage and also because of physical changes to the tail [32].

Lin et al. [33] discovered significantly lower post-thaw percent motility, motile sperm concentration, and cryosurvival rate, especially in a group of OAT patients. It is known that the loss of sperm quality is more significant in patients whose sperm parameters were poor to begin with [34]; however, there were also some differences found in the normozoospermic group. In the study by Stanic et al. [35], 63 sperm samples from normozoospermic patients were examined, and after cryopreservation, a reduction in motility was observed. The authors linked this reduction with the exposure to cryoprotectants, which is the most plausible explanation, but in a study by Kremer et al. [36], we can see the opposite opinion that exposure to CPAs does not affect motility.

Ozkavukcu et al. [18] reported that the main reason for decreased motility is the loss of vitality. They found a strong correlation between the increase in immotile spermatozoa and the decrease in viability. Their research was focused on motility, viability, and morphology, and each parameter worsened after freezing. In addition, Nur Karakus et al. [37] claimed that cryopreservation significantly reduces the quality of spermatozoa, mostly with respect to motility and viability. Moreover, after cryopreservation, between 25% and 75% of spermatozoa may become nonviable or lose their motility [38].

### 2.3. DNA Integrity and Acrosome Integrity

When we talk about damage from cryopreservation to motility, viability, and the plasma membrane, we can find several well-documented facts; however, the same is not true when discussing the damage cryopreservation causes to DNA. The integrity of sperm DNA is a highly important factor for the success of ART, so it must be a priority to focus on in further studies. Some research has been carried out, but studies show no agreement on whether cryopreservation affects the integrity of sperm DNA or not. Most studies focus on the integrity of the chromatin stability, sperm nucleus, and centrosome [20,34]. Differences between studies exist, most likely because of the use of different semen preparation techniques prior to cryopreservation, different freezing procedures, and the use of different tests to evaluate the integrity of the DNA. The tests used to assess DNA integrity are the TUNEL assay, Comet assay, and Acridine-staining assay. From the available references reviewed, we can confirm that researchers do not agree on the effect of cryopreservation on DNA integrity (Table 1).

In studies that have shown that cryopreservation causes DNA damage, researchers agree that there is a correlation between DNA damage and reduced fertilization rates, an increased risk of pregnancy loss, and some other reproductive outcomes [39,40,41]. In reproductive outcomes, we can find another important correlation between normal sperm heads and pregnancies. Part of the sperm head is a structure called an acrosome. The acrosome contains proteolytic enzymes that dissolve the ZP proteins at fertilization, so the acrosome must remain intact until it reaches the zona. Therefore, acrosome integrity is a very important parameter in the quality of sperm. Some studies described acrosome damage induced by cryopreservation; the problem mostly arises because of a change in membrane fluidity, which is a consequence of elevated levels of ROS [42,43]. Gomez-Torres et al. [20] investigated the damage cryopreservation causes to the structure of the acrosome and found that it is very susceptible to all changes that occur during the process. They claim that the freezing–thawing process provokes an acrosome reaction, which then results in the release of acrosomal enzymes such as in the initial sperm–zona binding. Low temperatures can also increase cytoplasmatic Ca^2+^ levels, which then cause the opposite reaction and enable the acrosome reaction [44]. Rahiminia et al. [45] studied whether a difference exists in acrosome damage between samples frozen via the slow-freezing method and samples frozen via vitrification They took 20 semen samples from healthy men and focused on acrosome and DNA integrity. Their results show that both exhibited a reduction in integrity after freezing and thawing, but more damage was observed in the vitrification group. The authors attributed this to the different drops in temperature that occurred during the different methods of freezing. It appears that spermatozoa can maintain their DNA and acrosome integrity better when the temperature drops slowly. Several authors [20,45] also exposed integrity of sperm chromatin as one of the key factors for human fertility and proper embryonic development. Fortunato et al. [46] measured chromatin condensation via aniline blue staining and found that chromatin integrity is highly affected by cryopreservation.

**Table 1:** Studies examining DNA damage induced by cryopreservation.

Method Used for DNA Damage Detection	Method and Cryoprotectant Used for Cryopreservation	Patients Included in the Study	Results	References
TUNEL	Freezing on liquid nitrogen vapor with glycerol as a cryoprotectant.	30 normozoospermic patients (>20 × 10^6^/mL and motility ≥50%)	There were no significant changes in DNA fragmentation observed.	Paasch et al., (2004) [47]
TUNEL	Freezing on liquid nitrogen vapor with glycerol as a cryoprotectant.	47 patients with oligozoospermia (<10 × 10^6^ sperm/mL) and 30 normozoospermic patients (>20 × 10^6^/mL and motility ≥50%)	An increase in apoptotic DNA fragmentation was observed in both groups, and there was no significant difference between groups.	de Paula et al., (2006) [48]
TUNEL	Freezing on liquid nitrogen vapor with glycerol as a cryoprotectant.	15 normozoospermic patients (>20 × 10^6^/mL and motility ≥50%)	A significant increase in DNA fragmentation after cryopreservation as well as decreases in sperm motility and viability.	Zribi et al., (2010) [49]
TUNEL	Programmable slow freezing and vitrification with glycerol as a cryoprotectant.	37 normozoospermic patients (>20 × 10^6^/mL and motility ≥50%)	A significant increase in DNA fragmentation for both methods, and a greater decrease in sperm motility after the vitrification method.	Tongdee et al., (2015) [50]
TUNEL	Freezing on liquid nitrogen vapor with glycerol as a cryoprotectant.	100 normozoospermic patients (>20 × 10^6^/mL and motility ≥50%)	Increased sperm DNA damage after cryopreservation.	Cankut et al., (2019) [51]
Comet	Freezing on liquid nitrogen vapor and vitrification with glycerol as a cryoprotectant.	38 normozoospermic patients (>20 × 10^6^/mL and motility ≥50%)	Cryopreserved spermatozoa were found to be unaffected by cryopreservation via both techniques, and their DNA integrity was comparable with that of fresh sperm.	Isachenko et al., (2004) [52]
Comet	Freezing on liquid nitrogen vapor with glycerol as a cryoprotectant.	166 patients (80 teratozoospermia, 32 normozoospermic, and 30 asthenoteratozoospermic, and 24 oligoasthenoteratozoospermic)	Increased sperm DNA damage in all groups, lower in a normozoospermic group. Higher levels of DNA damage in cryopreserved samples in comparison with fresh samples.	Ahmad et al., (2010) [53]
Comet	Freezing on liquid nitrogen vapor with glycerol as a cryoprotectant.	12 patients (6 normozoospermic, 3 asthenozoospermic, 1 oligozoospermic, 1 teratozoospermic, and 1 oligoasthenozoospermic)	Sperm DNA integrity was significantly negatively affected by cryopreservation.	Riel et al., (2011) [54]
Acridine Orange (AO) staining	Programmable slow freezing and freezing on liquid nitrogen vapor with glycerol as a cryoprotectant.	40 normozoospermic patients (>20 × 10^6^/mL and motility ≥50%)	A post-thaw increase in sperm DNA damage; programmable slow freezing provided superior results than freezing on liquid nitrogen vapor.	Somsin et al., (2007) [55]

## 3. Factors Causing Cryodamage

### 3.1. Oxidative Stress

Unfortunately, the studies agreeing that cryopreservation causes DNA damage do not agree on the mechanism through which the damage is caused. In the articles reviewed (Table 2), most attention was paid to three mechanisms that are supposed to be the culprits of DNA damage. The first mechanism is oxidative stress, followed by abortive apoptosis and chromatin remodeling. However, DNA damage usually occurs due to a combination of all three mechanisms [56]. It is common knowledge that managed levels of ROS are important in normal processes such as acrosome reaction, capacitation, and other processes needed for fertilization, but it becomes difficult when ROS overcome defense systems and destroy this balance, what may occur during cryopreservation [57]. The plasma membrane of human sperm, as previously mentioned, is full of fatty acids which are vital for the membrane. However, these fatty acids are very susceptible to attack by free radicals because of their double-bonded nature, making spermatozoa very susceptible to any harm that comes from processes involving oxidative stress. Likewise, ROS play an important role in sperm DNA fragmentation because transition metals such as iron and copper encourage the capacity of ROS to attack the DNA in the nucleus and mitochondria [58].

In summary, impaired sperm quality is caused by ROS via two mechanisms: damaging the sperm DNA straightforwardly and by causing lipid peroxidation of the sperm plasma membrane, which leads to the formation of toxic products and consequently reduces sperm motility and the ability of the spermatozoa to adhere to the [56].

Despite contradictory results, we can conclude that oxidative stress participates as one of the primary factors causing poor semen quality after cryopreservation, so we must focus on reducing oxidative stress. This can be achieved by either minimizing the levels of the sources of ROS production or by neutralizing ROS [59].

**Table 2:** Studies examining the correlation between DNA damage and increased levels of ROS.

Method Used for DNA Damage Detection	Method and Cryoprotectant Used Cryopreservation	Patients Included in the Study	Results	References
Flow cytometry	Programmable slow freezing with glycerol as a cryoprotectant.	18 normozoospermic patients (>20 × 10^6^/mL and motility ≥50%)	Levels of ROS were increased after cryopreservation.	Wang et al., (1997) [60]
Flow cytometry	Programmable slow freezing with glycerol as a cryoprotectant.	60 patients (34 with abnormal semen results and 26 with normal semen results)	The process of cryopreservation resulted in an increase in DNA fragmentation. The dominant pathway to DNA fragmentation during cryopreservation is the ROS pathway.	Thomson et al., (2009) [61]
Flow cytometry	Freezing on liquid nitrogen vapor with glycerol as a cryoprotectant.	30 normozoospermic patients (>20 × 10^6^/mL and motility ≥50%)	The levels of ROS detected via flow cytometry increased significantly compared with the fresh control group.	Li et al., (2010) [62]
Flow cytometry	Freezing on liquid nitrogen vapor with glycerol as a cryoprotectant.	15 normozoospermic patients (>20 × 10^6^/mL and motility ≥50%)	They found no relationship between DNA fragmentation and ROS levels; they suggest cryopreservation-induced DNA damage happens through other pathways.	Zribi et al., (2010) [49]
Flow cytometry	freezing on liquid nitrogen vapor and vitrification with glycerol as a cryoprotectant	49 patients of infertile couples undergoing routine semen analysis	Both cryopreservation methods induced higher levels of ROS production. Results with the vitrification method were poorer than results achieved via vapor freezing.	Arciero et al., (2021) [63]

### 3.2. Osmotic Stress

To maintain rates of cryosurvival that are as high as possible, it is necessary to add cryoprotectants to the spermatozoa before freezing. However, adding a CPA leads to osmotic stress, which can be very toxic for spermatozoa. During the freezing process, ice crystals start to emerge, and there is less water left in which the solutes dissolve, so the concentrations of extracellular solutes and CPAs increase, creating a hyperosmotic environment for the cells that can affect different cell mechanisms related to cell viability. This causes the so-called “solution effect”, which includes influencing the cell membrane permeability, increasing cellular dehydration, and changing the pH [64]. Critser et al. [65] and Penninckx et al. [66] both confirmed that the cells’ osmotic environment may cause cryoinjuries and is induced by adding a CPA before freezing and removing the CPA during thawing. To limit osmotic cryoinjury, we need to know the osmotic tolerance of the cells and then choose the right cryopreservation technique and CPA for spermatozoa.

### 3.3. Temperature Stress

The common term used for temperature stress is “cold shock”, and it can cause damage to intracellular organelles, change membrane permeability, and induce a loss of motility. All this damage can be assigned to a phase change in lipids and to a change in the functional state of the membrane [59]. De Leeuw et al. [67] found out there were morphological changes in the membranes of bull and boar sperm samples due to a lipid phase transition (from fluid to gel) at specific temperatures, and there were also changes in the membranes’ viscosity in the same temperature range. The most important and problematic consequence of cold shock is the formation of ice crystals that are pure crystalline water and have the ability to dissolve solutes. These solutes then concentrate [68], resulting in the osmotic stress described above.

## 4. Cryoprotectants and Antioxidants

Cryoprotectants are low-molecular-weight essential substances used for minimizing the stress produced by freezing and thawing, especially the stress induced by ice formation. They adjust the cell environment (intra- and extracellular) and prevent ice formation by lowering the freezing point of the solution and keeping the extracellular environment in the liquid phase at any temperature (even below zero), increasing the total concentration of all components present in the cryoprotective medium [69,70]. However, they are only helpful at appropriate concentrations: if the concentration is too high, they can be toxic to cells, so the composition of the cryoprotective medium is very important in all freezing techniques. All CPAs are highly water soluble and work straight to the membrane, yet they have different chemical compositions, and we can divide them into two classes: permeating CPAs and non-permeating CPAs [70]. The difference between these two groups is whether they cross the membrane: permeating CPAs cross the membrane, and non-permeating CPAs do not.

The permeating CPAs used most often are glycerol, ethylene glycol, dimethyl sulfoxide (DMSO), and 1,2-propanediol (PROH). These CPAs move easily across the membrane and create an osmotic gradient which causes water to leave the cell and the cell to shrink. They also lower the freezing point and provide intracellular protection. Non-permeating CPAs have a high molecular weight which disables them from crossing the cellular membrane, so they generate an osmotic gradient outside the cell that causes water to leave the cell. These CPAs can be divided into disaccharides (sucrose and trehalose), polysaccharides (maltodextrin), and proteins (albumin) [71]. Aside from limiting ice formation, CPAs have one more important function when the temperature becomes very low: they enter a glassy state which functions as a matrix. In this so-called matrix, molecular reactions are slowed, which that stabilizes cells for long-term storage [72]. Cryoprotective media for human sperm contain the most commonly used cryoprotectant—glycerol—and several other nutrients, such as sugars, lipids, proteins, and salt. Glycerol’s primary task is to protect the spermatozoa against thermal shock. This is achieved by influencing the cell membrane by acting on the stability and permeability of the lipid bilayer and its structure. This changes the cell’s metabolism and associations with surface proteins [70]. The other important component of CPAs are sugars, which optimize the osmotic gradient and supply the spermatozoa with energy. For decades, researchers were using egg yolk to freeze mammalian sperm because it supposedly improved the fluidity of the membrane and protected the integrity of the sperm [73,74,75]; however, it has an undefined composition, so they had to find alternatives. Liposomes were identified as an appropriate alternative, and cryoprotective media is currently egg-yolk-free and contains only chemically defined components, including glycerol and sucrose as the cryoprotective agents [76,77]. Hossain and Osuamkpe [78] were investigating the solo use of sucrose, which demonstrates some good properties, because sugars increase the glass transition temperature and allow storage at lower temperatures; however, their efficiency requires more research. Sherman [79] was looking into adding only glycerol for cryopreservation, but it did not provide the best results. His study showed that glycerol alone damages the plasma membrane, nucleus, and acrosomal integrity. He also used dimethyl sulfoxide (DMSO) and propanediol (PROH), but no success was achieved. Nowadays, DMSO is regularly used for preventing the formation of water crystals, the same as glycerol [80].

As previously mentioned, cryopreservation has some harmful effects on spermatozoa, and improvements have been made year by year. One of the topics highlighted at present is the addition of antioxidants to the freezing media. Antioxidants are molecules that are able to reduce oxidative processes by eliminating released free radicals [81]. This implies a decrease in the negative effects of ROS and an improvement in the quality of post-thaw semen. The antioxidants used in studies are vitamin E, glutathione (GSH), sericin, superoxide dismutase (SOD) and/or catalase (CAT), vitamin C, melatonin (MLT), selenium (Se), and some natural herbs—genistein, rosemary, curcumin, green tea extract, and oregano extract (Table 3).

## 5. Cryopreservation Techniques

In sperm cryopreservation, two standard freezing methods developed in the middle of the 20th century are still used today: programmable slow freezing and freezing on LN_2_ vapors. Despite the successes of these empirical methods, there has been some questioning of new methods that are faster, cheaper, and simpler. One of these promising methods is vitrification, which is used only for freezing embryos and oocytes at present. Studies on the vitrification of human sperm provide very conflicting results, quite possibly because human spermatozoa are perhaps more susceptible to osmotic changes than other reproductive tissues and because heat transfer in sperm cells is too slow, increasing the risk of crystallization [88,89].

Methods based on a slow cooling rate have roughly the same protocols, which involve adding CPAs before freezing, freezing the cells, thawing the cells, and removing the CPAs after thawing. The most critical temperature range for sperm cells is between −10 °C and −60 °C, and they go through this range twice: first via freezing and second via warming. Slow freezing methods are based on slowly lowering the temperature, especially in the zone that can be lethal for cells. Also, the focus is on the balance between the growing concentration of dissolved substances and the formation of ice crystals [90]. Programmable slow freezing is based on dehydration, and the protocol takes 2–3 steps and 2–4 h to complete. The temperature drop (0.5–1 °C/min) is controlled by the machine, and it freezes the plastic straws before they are plunged into LN_2_. During freezing on LN_2_ vapor, the plastic straws are filled with the sperm sample and a cryoprotective medium, in a 1:1 proportion, left at room temperature for 10 min, placed at approximately 5 cm above the LN_2_ for 30 min, and then plunged into the LN_2_ for storage [91]. Opposite to slow freezing is the vitrification method, which is a rapid method. In vitrification, the cells are cooled at an extremely high rate and enter a vitrified state via an elevation in viscosity [92]. A semen sample mixed with a freezing medium is loaded into straws or another type of device, such as a cryoloop, and plunged directly into the LN_2_ [93]. During vitrification, not only are there rapid and high cooling rates but high concentrations of CPAs are also required; this seems to be the main problem for sperm vitrification because spermatozoa are osmotically fragile and are not capable of tolerating high concentrations of CPAs at high speeds [94]. Vitrification is a very simple method compared to slow freezing; the problem with vitrification is found in its inability to preserve large volumes of sperm, and since regular vitrification methods provide very low spermatozoa survival rates, many researchers stopped investigating this method of freezing for some time. However, it was later suggested that there may be a means of vitrifying spermatozoa without toxic concentrations of CPAs. Isachenko et al. [19] believed that with a small sample size and a low concentration of permeable CPAs, vitrification may produce a positive output. They used 1% of human serum albumin (HSA) instead of permeable CPAs to create a suspension of human spermatozoa. They placed 20 µL drops of the suspension onto a copper loop and directly plunged it into LN_2_. Their research showed some promising results in comparison with conventional slow-freezing methods: motility and viability were both higher with the use of HSA and sucrose. Vutyavanich et al. [95] vitrified human sperm in straws with a minimum concentration of trehalose in a vitrification medium, and the post-thaw motility and other parameters were better than results achieved with a slow-freezing method. However, the vitrification of human spermatozoa is still not commonly used. On one hand, it is superior to slow freezing in many areas, but on the other hand, much more research must be carried out for it to finally become a routine practice in cryopreservation.

### 5.1. Comparison of Slow Freezing Vs. Vitrification

As we can see, sperm cryopreservation is a well-used technique in ART and plays an important role in several areas to help patients save their genetic material. However, the cryoinjuries that may happen during cryopreservation have negative effects on cell functions, so it is important to understand the modifications that are present during the cryopreservation process and then optimize the freezing technique. As described above, there are many techniques available, the majority of which are standard procedures that have been used for quite some time now and are still very useful, but some progress must be made to achieve even better post-thaw results. A certain number of new procedures have been introduced by researchers in recent years, but there are still different opinions about them. The most frequent question in studies is, which sperm cryopreservation technique provides better post-thaw results? The technical aspects of the methods have been clarified by now, but there is still no standard method that optimizes the recovery of sperm parameters affected by freezing and thawing in all aspects. Answers to this question differ, and the optimal rate of temperature decrease during freezing has not been determined yet, so there is no definitive conclusion in the comparison of slow freezing and vitrification. In addition, there are differences between cryoprotective agents and between the devices used for cryopreservation, and no definite conclusion has been reached as to which ones are the most effective. Hosseini et al. [96] compared freezing on LN_2_ vapor and vitrification with a Cryotech device, using sperm from normozoospermic patients. They focused on chromatin condensation, DNA fragmentation, motility, and viability before and after cryopreservation. Their results showed higher sperm motility and viability in the samples frozen on vapor than via vitrification, but DNA integrity was not found to be significantly different between these two methods. Similar results for motility were achieved by Isachenko et al. [52], who also compared the cryopreservation of human sperm via vitrification and freezing on vapor and observed the same sperm parameters as Hosseini and her group. An average reduction in motility of 40% compared with the control group was found, but there was no significant difference between the vitrification and vapor freezing. DNA integrity was unaffected by freezing, which indicates that the small size of the spermatozoon head may be beneficial for intracellular vitrification without ice formation. They mentioned there is the possibility of cryoprotectant-free cryopreservation of human sperm to avoid the toxic effects of CPAs. In a study by Riva et al. [97], DNA integrity and sperm motility after slow freezing and vitrification were taken into consideration. They found that motility was higher in samples frozen via the ultra-rapid method than those frozen via slow freezing, and levels of DNA fragmentation were higher in the group of samples frozen via slow freezing. A higher level of post-thaw motility was mentioned in research by Li et al. [88], but they achieved the opposite result, finding that motility and vitality were higher after freezing via the slow-freezing method than vitrification, but vitrification provided better results with respect to morphology. In other studies, researchers compared not only the cryopreservation method but also the cryopreservation media used. Nallella et al. [98] used three different media (TYB, Sperm Freezing Medium, and Enhance sperm freeze) to freeze samples on vapors and then observed sperm motility. The highest sperm motility was noted in the TYB medium, and there was no difference in sperm motility between the samples frozen via slow freezing or vitrification.

The studies we examined did not share the same opinion as to which method is superior: some authors prefer empirical methods, and others prefer vitrification. Vitrification is cheaper, it takes less time, and it does not require any post-thaw processes, but CPAs are toxic for sperm cells, and no large volume of sperm samples can be vitrified [99]. Slow freezing requires a freezing medium and post-thaw processing to separate the permeable CPA from sperm, which costs more and takes more time, but positive aspects of slow freezing are the use of less-toxic CPAs and that a large volume of sperm can be frozen. We cannot conclude which method provides better post-thaw results. Slow freezing techniques are still very popular, and storage in LN_2_ is a safe environment for samples, but it is clear that researchers are finding new ways to store higher-quality samples of sperm and improve pregnancy outcomes. Some of the new techniques discussed in articles that have the potential to develop into well-used methods are lyophilization [100], the storage of sperm in zona pellucida [101], cryoloops [102], and storage in ICSI pipettes [15]. Lyophilization means removing all water molecules from a sample of human sperm, which is achieved by cooling it to the temperature at which substances go directly from a solid state to a gaseous state. A negative effect of this method is that all spermatozoa undergoing lyophilization are immotile after the procedure, but the DNA damage is less than when vapor freezing occurs. In addition to less DNA damage, a positive aspect of this method is its storage at 4 °C because samples can therefore be transported at room temperature [103]. The storage of sperm in human or animal zona pellucida includes many different protocols, from laser-assisted techniques for making holes to remove the cytoplasmic contents to the insertion of the best sperm cells into the zona pellucida [101]. The samples are then frozen via the slow-freezing method and after thawing, the sperm parameters (motility, viability, and DNA integrity) recover very well. With a cryoloop, it is possible to catch small volumes of semen samples because of capillary action, which can be useful for vitrification. For the storage of small volumes of samples during slow freezing, ICSI pipettes can also be used although they break easily, which increases the risk of contamination. The storage of small volumes is also possible by mixing microdroplets of sperm with CPA, which are then cooled on dry ice or LN_2_ vapors [103].

### 5.2. Cryopreservation Affects Spermatozoa on a Molecular Level

**Figure 1:**
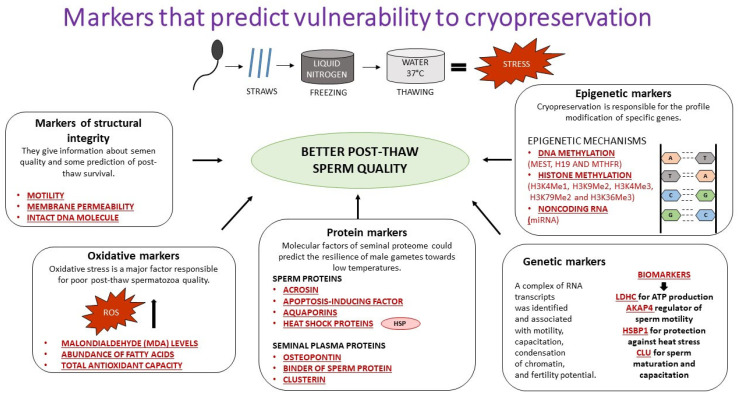
Markers that predict vulnerability to cryopreservation.

Even though cryopreservation is very significant worldwide, a complete understanding of all the markers predicting cryoresistance and the markers predicting vulnerability to cryopreservation is still lacking. The markers that can help us understand sperm physiology during the freezing and thawing processes can be split into five groups, (1) protein markers, (2) oxidative markers, (3) markers of structural integrity, (4) genetic markers, and (5) epigenetic markers (Figure 1) [104].

Cryopreservation causes structural alterations (changes in membrane permeability, changes in motility, acrosome damage, etc.) [105,106] and molecular alterations such as DNA fragmentation, the degradation of mRNA, alterations to DNA methylation, changes in the DNA/protamine complex, and alterations to miRNA cargo [107,108,109,110,111]. Injuries on the molecular level that are generated by cryopreservation are perhaps larger than injuries on the structural level.

Studies that are relevant to this topic were mostly conducted on the semen of mammals such as boars, stallions, mice, and horses; until now, very few studies have been conducted on human sperm. The majority of the damage inflicted upon spermatozoon molecules is usually demonstrated through the fertilizing capacity of the cryopreserved sperm, which is related to the processes that take place during fertilization [107]. One of the key processes happening during fertilization is the release of messenger RNAs (mRNA) inside the oocyte by the spermatozoon [112]. Due to the translation processes and synthesis of proteins by the oocyte, these genes may also be relevant for early embryo development [113]. As mentioned above, cryopreservation affects mRNA molecules, and the problem we face with spermatozoa is the insufficiency of replacing the damaged mRNA because spermatozoa are so-called transcriptionally “silent” cells [114]. The effect of cryopreservation on human sperm mRNA was investigated by Valcarce et al. [115], who analyzed the effect on two groups of transcripts after vitrification and slow freezing. One group contained male fertility markers (protamine 1, protamine 2, BCL2-interacting killer, and FSHb polypeptide), and the other group contained pregnancy success markers (activin A receptor type II-like 1, adducin 1 alpha, androgen receptor, aryl-hydrocarbon receptor nuclear translocator, and endothelial PAS domain protein). They claimed that several transcripts in human spermatozoa disappear after cryopreservation, which was also mentioned before by Flores et al. [109], and their hypothesis was that cryopreservation does affect the stability of transcripts to such an extent that they degrade. In their results, we can see that cryopreservation remarkably affects two of the five mRNAs in a group of pregnancy success markers and four of the five mRNAs in a group of male fertility markers, and there was no difference between the slow freezing results and vitrification results. In all, their results show that cryopreservation can influence spermatozoa on a molecular level, which changes the success of fertilization and accurate early embryo development. The identification of components that affect successful fertilization capacity was also a priority in research conducted by Bogle et al. [116]. They wanted to gain insight into the proteomic changes induced by cryoprotectants during slow freezing and vitrification to understand the mechanisms of cryoinjury in spermatozoa. They quantified changes in the sperm proteome and found meaningful changes which may be the consequences of protein degradation caused by a cryoprotectant or translocation to other cellular parts. Their results show that an abundance of proteins may be influenced by different laboratory procedures; however, it does not matter if the sample was collected from a human [112,116], boar [117], or fish [118]. All the studies mentioned above share a common opinion that cryopreservation changes proteins, which play major roles in sperm motility, capacitation, fertilization, membrane permeability, and sperm metabolism. One of the molecules cryopreservation also has an impact on is microRNA. Experimental research by Rahbar et al. [119] showed that in human spermatozoa and testis, microRNAs are found and play a functional role in spermatogenesis and cryopreservation. There is a family of so-called cold-modulated miRNAs which cause resistance to freezing by inhibiting the ATP metabolism to adjust the cell to different levels of energy consumption and regulate freezing stress [120]. It may also be possible that miRNAs effect the expression of mRNA during cryopreservation [121] and influence fertilization results [120,122,123].

## 6. Conclusions

In summary, sperm cryopreservation is an essential technique, but it has many negative effects on sperm parameters because it causes a major decline in DNA integrity, membrane viability, motility, and viability. With all articles reviewed, there is no certain conclusion with respect to prioritizing a specific method, but we can assume that there is a bright future ahead for optimizing cryopreservation methods to a point at which we can achieve better fertility outcomes and higher pregnancy rates. Researchers are discovering new means of improving post-thaw sperm parameters, such as adding antioxidants and focusing on proteomics and genome sequencing to search for proteins as potential biomarkers associated with screening for fertilization potential and which may also be useful for indicating cryostress [124]. Despite everything, the most promising method is vitrification due to of all the above-mentioned benefits; moreover, it does not technically require the use of a permeating CPA and therefore does not cause lethal osmotic shock to spermatozoa. However, more research must be performed on larger volumes of sperm samples.

## Figures and Tables

**Table 3:** Studies investigating the effect of antioxidants supplemented with freezing media on cryopreserved spermatozoa.

Antioxidant Used	Method and Cryoprotectant Used for Cryopreservation	Patients Included in the Study	Results	References
Vitamin E	Freezing on liquid nitrogen vapor with glycerol as a cryoprotectant.	59 patients with asthenozoospermia and 38 normozoospermic patients (>20 × 10^6^/mL and motility ≥50%)	Supplementing the cryoprotectant with VE significantly enhanced the total motility and progressive motility in normozoospermic as well as asthenozoospermic samples.	Kalthur et al., (2011) [82]
Melatonin	Freezing on liquid nitrogen vapor with glycerol as a cryoprotectant.	43 normozoospermic patients (>20 × 10^6^/mL and motility ≥50%)	The results show that the supplementation of melatonin significantly increased motility and viability and decreased levels of intracellular ROS.	Karimfar et al., (2015) [83]
Sericin	Freezing on liquid nitrogen vapor with glycerol as a cryoprotectant.	51 normozoospermic patients (>20 × 10^6^/mL and motility ≥50%)	The addition of sericin significantly increased sperm viability and total motility and decreased DNA fragmentation.	Aghaz et al., (2018) [84]
Oregano Extract (*Oregano vulgare*)	Freezing on liquid nitrogen vapor with glycerol as a cryoprotectant.	20 normozoospermic patients (>20 × 10^6^/mL and motility ≥50%)	The total motility was significantly increased in frozen–thawed spermatozoa in comparison with the control group. The percentage of vital spermatozoa was also significantly higher.	Shiri et al., (2020) [85]
Green Tea	Freezing on liquid nitrogen vapor with glycerol as a cryoprotectant.	45 normozoospermic patients (>20 × 10^6^/mL and motility ≥50%)	They found that supplementing the sperm-freezing media with GTE had a significant protective effect on human sperm motility and DNA integrity, but there was no significant change in the ROS level.	Alqawasmeh et al., (2021) [86]
Curcumin	Freezing on liquid nitrogen vapor with glycerol as a cryoprotectant.	23 normozoospermic patients (>20 × 10^6^/mL and motility ≥50%)	In the curcumin group, progressive motility, sperm chromatin condensation, and DNA integrity significantly increased after the thawing process when compared with the control.	Karakus et al., (2021) [37]
Curcumin	Freezing on liquid nitrogen vapor with glycerol as a cryoprotectant.	60 normozoospermic patients (>20 × 10^6^/mL and motility ≥50%)	The results showed that curcumin supplementation in a freezing medium was protective for human sperm parameters (increased total motility) and sperm DNA (decrease in DNA fragmentation).	Santonastaso et al., (2021) [87]

## Data Availability

Not applicable.
